# Uræmic Convulsions in a Pug

**Published:** 1881-07

**Authors:** 


### URÆMIC CONVULSIONS IN A PUG—AUTOPSY.

EDITOR OF JOURNAL OF COMPARATIVE MEDICINE:—

The occurrence of convulsions in dogs that have been closely bred is, I believe, of quite frequent occurrence. As any especial study of the pathology of these convulsions is correspondingly rare, I send a report of the following case:

The dog in question was a full-blooded pug, eighteen months old. It had previously been very healthy, except for a slight amount of mange. Its mistress had treated this, without success, by warm soap-suds, which she applied every Sunday, to the neglect of her religious duties.

Its diet had been oatmeal and milk, with an occasional bone and "extras." Under this it had become fat.

On April 14th the dog was taken with a kind of delirium, running wildly about the yard. Convulsions soon set in, tonic and clonic in character. The pupils were widely dilated, and the animal frothed at the mouth. After a time he was unable to run about. Cold water was thrown upon him, *secundum artem*, by a frightened domestic, when the convulsions began; but this seeming to do no good, he was placed in hot water, with mustard, and a minim of Magendie's solution injected hypodermically. This produced no relief, and the dog soon died, respiration ceasing first.

AUTOPSY.—The brain was slightly congested, but showed no other change. Lungs somewhat congested; heart normal.

The abdominal viscera were normal, except the kidneys. These were very red and congested, the Malpighinan bodies showing plainly. Under the microscope, the evidences of an acute diffuse nephritis seemed to be quite well marked.

The nervous system of such dogs as the pug is finely organized, and easily thrown out of balance. It is probable that the morbid change in the kidneys threw sufficient poison into the blood to excite the convulsions.

C. L. D.

NEW YORK, June 9th.

